# Microbial production host selection for converting second-generation feedstocks into bioproducts

**DOI:** 10.1186/1475-2859-8-64

**Published:** 2009-12-04

**Authors:** Karl Rumbold, Hugo JJ van Buijsen, Karin M Overkamp, Johan W van Groenestijn, Peter J Punt, Mariët J van der Werf

**Affiliations:** 1 Team Microbial Production Processes, TNO Quality of Life, PO Box 360, 3700 AJ Zeist, The Netherlands; 2 Current address: School of Molecular and Cell Biology, University of the Witwatersrand, Johannesburg, South Africa

## Abstract

**Background:**

Increasingly lignocellulosic biomass hydrolysates are used as the feedstock for industrial fermentations. These biomass hydrolysates are complex mixtures of different fermentable sugars, but also inhibitors and salts that affect the performance of the microbial production host. The performance of six industrially relevant microorganisms, i.e. two bacteria (*Escherichia coli *and *Corynebacterium glutamicum*), two yeasts (*Saccharomyces cerevisiae *and *Pichia stipitis*) and two fungi (*Aspergillus niger *and *Trichoderma reesei*) were compared for their (i) ability to utilize monosaccharides present in lignocellulosic hydrolysates, (ii) resistance against inhibitors present in lignocellulosic hydrolysates, (iii) their ability to utilize and grow on different feedstock hydrolysates (corn stover, wheat straw, sugar cane bagasse and willow wood). The feedstock hydrolysates were generated in two manners: (i) thermal pretreatment under mild acid conditions followed by enzymatic hydrolysis and (ii) a non-enzymatic method in which the lignocellulosic biomass is pretreated and hydrolyzed by concentrated sulfuric acid. Moreover, the ability of the selected hosts to utilize waste glycerol from the biodiesel industry was evaluated.

**Results:**

Large differences in the performance of the six tested microbial production hosts were observed. Carbon source versatility and inhibitor resistance were the major discriminators between the performances of these microorganisms. Surprisingly all 6 organisms performed relatively well on pretreated crude feedstocks. *P. stipitis *and *A. niger *were found to give the overall best performance *C. glutamicum *and *S. cerevisiae *were shown to be the least adapted to renewable feedstocks.

**Conclusion:**

Based on the results obtained we conclude that a substrate oriented instead of the more commonly used product oriented approach towards the selection of a microbial production host will avoid the requirement for extensive metabolic engineering. Instead of introducing multiple substrate utilization and detoxification routes to efficiently utilize lignocellulosic hydrolysates only one biosynthesis route forming the product of interest has to be engineered.

## Background

Industrial (or white) biotechnology is increasingly being applied for the production of a large number of chemicals such as bioethanol, citric acid, amino-acids and 1, 3-propanediol. It is anticipated that within a few years products produced by biotechnology will contribute to 10% of total sales of the chemicals industry [1]. To reach this target, it is not only required that the production of such products is technical feasible, but also that their cost price can compete with the same compound being produced from petrochemical resources.

In industrial biotechnology, substrate (feedstock) costs are by far the highest cost factor in the production of (bulk-)chemicals, representing 40 - 60% of the total costs [2]. Therefore, there is an increasing interest in using cheap lignocellulosic biomass streams as the feedstock for industrial biotechnology processes. Not only are lignocellulosic (second generation) feedstocks cheaper compared to first generation feedstocks, their use does also not compete with the supply of food and feed and results in an overall more environmentally friendly bioprocess [3].

The selection of a microbial production host for an industrial biotechnology process is primarily determined by its potential to efficiently produce the product of interest. Moreover, there is a preference for microorganisms that are well characterized, genetically accessible and therefore have the potential to become microbial production platforms. However, second generation feedstocks are much more complex than first generation feedstocks. They consist of a mixture of different fermentable sugars (i.e. glucose, xylose, arabinose, galactose, mannose, etc.) and - depending on the pre-treatment and hydrolysis process applied to convert the lignocellulose into the fermentable sugars - different inhibitors and high salt concentrations are present in these pretreated feedstocks [4]. Therefore, production hosts are being adapted to be able to utilize second generation feedstocks. An example is the yeast *Saccharomyces cerevisiae*, the microorganism most widely applied for the production of bioethanol. Wild-type *S. cerevisiae *is not able to utilize the pentoses xylose and arabinose that are abundantly present in lignocellulosic hydrolysates. Moreover, this yeast is quite sensitive to inhibitors formed during the thermal pretreatment of lignocellulose, such as furfural and hydroxymethylfurfural (HMF). Therefore, extensive metabolic engineering in combination with evolutionary engineering of *S. cerevisiae *has been performed, and now strains are available that are able to utilize xylose [5] and arabinose [6] or that are more resistant against furfural and HMF [7,8].

In view of the complexity and variety of second generation feedstocks, and the extensive metabolic/evolutionary engineering required to adapt first generation production hosts to second generation feedstocks, it might therefore be more efficient to change the host selection approach from product-oriented to substrate-oriented. In this study, wild-type strains of six commonly applied industrial production hosts, i.e. 2 bacteria, 2 yeast and 2 fungi, were compared with respect to their natural ability and general suitability to utilize second generation feedstocks.

## Results

### Utilization of different carbon sources

To be able to look at their natural performance on feedstocks, the microbial strains used in this study were wild-type strains. Additionally, microbes of which the genome sequence and some basic features with respect to genetic accessibility and growth characteristics are known were chosen in order to facilitate subsequent follow-up research by metabolic engineering.

The six microorganisms were tested for their ability to utilize monosaccharides abundantly present in lignocellulosic hydrolysates and on glycerol (Table 1). Of these six microorganisms, only *E. coli *was able to utilize all these compounds as sole source of carbon and energy. The other microorganisms were not able to readily utilize one or two of the substrates tested.

**Table 1:** Carbon source versatility

	Glucose	Xylose	Arabinose	Mannose	Galactose	Glycerol
*E. coli*	+	+	+	+	+	+

*C. glutamicum*	+/-	-	-	+/-	+/-	+/-

*S. cerevisiae*	+	-	-	+	-	+/-

*P. stipitis*	+	+	-	+	+	+

*T. reesei*	+	+	-	+	-	-

*A. niger*	+	+	+	+	-	+

+ good growth

+/- moderate growth

- no or very slow growth

Subsequently, the performance of these microorganisms with respect to growth and carbon source uptake rate as well as biomass yield was established by cultivating the six microorganisms under controlled conditions in a fermentor on glucose, xylose and glycerol (Table 2). These three carbon sources were chosen because they are most abundant in second generation feedstocks. Growth rate and carbon source uptake rate showed a clear correlation. However, the biomass yield was independent of these two parameters. Overall the bacteria studied grew faster than the yeasts, which in turn grew faster than the filamentous fungi. Apart from the type of organism also the carbon source had an influence on growth rate, for instance *S. cerevisiae *showed a 16 fold higher growth rate on glucose compared to glycerol. Two of the microorganisms, i.e. *P. stipitis*, and *A. niger *grew clearly faster on xylose than on glucose.

**Table 2:** Performance of the six microorganisms on three carbon sources in controlled fermentations.

	Carbon source	Growth rate (1/h)	**Carbon source uptake rate**^**1**^ (g/g/h)	**Biomass yield**^**2**^**(g/g)**
*E. coli*	Glucose	0.49	1.56	0.28

	Xylose	0.50	1.71	0.20

	Glycerol	0.40	0.88	0.36

*C. glutamicum*	Glucose	0.33	0.79	0.31

	Xylose	-	-	-

	Glycerol	0.18	1.09	0.12

*S. cerevisiae*	Glucose	0.20	2.52	0.09

	Xylose	-	-	-

	Glycerol	0.01	0.01	0.6

*P. stipitis*	Glucose	0.10	0.34	0.14

	Xylose	0.40	2.16	0.10

	Glycerol	0.11	0.46	0.14

*T. reesei*	Glucose	0.02	0.08	0.22

	Xylose	0.01	0.04	0.18

	Glycerol	-	-	-

*A. niger*	Glucose	0.03	0.20	0.34

	Xylose	0.07	0.13	0.41

	Glycerol	0.03	0.11	0.24

^1 ^The rate was determined for the biomass dry weight at mid-log phase.

^2 ^Biomass dry-weight value was used at the time point when the carbon source was depleted.

*S. cerevisiae *exhibits strong variations in fermentation parameters, depending on the carbon source. The other organisms, particularly fungi, show less variation when grown on different carbon sources. In view of the complexity of real-life feedstocks, utilization of a broad range of substrates resulting in high yields is an important criterion in the selection of industrial production hosts. Moreover, growth and carbon source uptake rate are measures for an efficient cellular metabolism that can be used to compare production hosts for their suitability for lignocellulose utilization.

### Resistance against inhibitors

The production of fermentable sugars from lignocellulose feedstock always results in by-product formation [4]. The type and amount of inhibitors found in lignocellulose hydrolysates depends on the hydrolysis method. In our study we have applied the two most commonly used methods: Mild acid thermal pretreatment followed by enzymatic hydrolysis [9] and concentrated acid hydrolysis [10,11]. Together with acetic acid, which is derived from hemicellulose in the plant cell wall, the sugar degradation products furfural and 5-hydroxymethyl furfural (HMF) are the inhibiting compounds found at the highest concentrations in hydrolysates. High concentrations of magnesium sulphate are found in hydrolysates resulting from concentrated acid hydrolysis [11]. Crude glycerol from biodiesel production contains up to 10% (w/w) sodium chloride [12], while in cellulosic biorefineries that recycle water the chloride concentration can become high as well. All six test organisms were grown in the presence of increasing concentrations of these inhibitors and growth parameters were compared (Table 3).

**Table 3:** Resistance of the microorganisms studied to inhibitors present in feedstock hydrolysates

Production host	Furfural	HMF	Acetate	NaCl	**MgSO**_**4**_	Growth at pH4
*E. coli*	1	2	5	40	80	-

*C. glutamicum*	0.5	2	10	40	> 80	-

*S. cerevisiae *Bioscreen*	1	1	5	20	> 80	+/-

*S. cerevisiae *Micro-Oxymax*	n.d.	1	n.d.	20	n.d.	n.d.

*P. stipitis*	1	2	5	40	> 80	+/-

*T. reesei*	1	5	20	100	> 80	+

*A. niger*	1	5	20	100	> 80	+

The concentration (g/l) of the inhibitor is shown at which inhibition results in <50% of the growth rate/CO_2 _production rate without inhibitor.

*In order to check correlation of the methods, *S. cerevisiae *was evaluated using both the Bioscreen and Micro-Oxymax method (see Methods).

n.d. = not determined

+ good growth

+/- moderate growth

- no or very slow growth

Due to their filamentous growth, growth parameters for both fungi could not be determined using the Bioscreen approach, therefore for these organisms growth was determined by monitoring the CO_2 _production in the gas phase. To allow further comparison of the data between the two methods, *S. cerevisiae *resistance to HMF and sodium chloride was analyzed by both methods. As can be seen in Table 3, good correlation of both methods was found.

In general, fungi were more resistant to the tested inhibitors than the other host organisms. Furfural strongly inhibits all six test organisms at low concentrations (0.5 - 2 g/l). However, the growth of filamentous fungi is hardly affected in the presence of high concentrations of HMF, acetic acid and sodium chloride when compared to the other microorganisms studied. In the relevant concentration range magnesium sulphate does not affect the growth of the test organisms. In addition to inhibitor resistance we also tested pH sensitivity of the growth of the selected organisms. pH sensitivity is an undesired trait, as in order to overcome contamination and allow the use of non-sterile feedstocks, most fermentations are preferably carried out at low initial pH [13]. As shown in Table 3 both bacterial strains are sensitive to a culture pH below 4.

### Production of extracellular metabolites

Samples from controlled fermentations were taken at late log-phase and subjected to analysis using our metabolomics platform [14-16]. Table 4 gives an overview of the most abundant metabolites produced by these microorganisms. As a cut-off for our comparison a metabolite concentration of 0.1 g/l fermentation broth was taken. On average, *E. coli *and *S. cerevisiae *produce metabolites in higher concentrations and wider variety than the other test organisms due to their strong overflow metabolism [17]. In this study, the growth medium used was largely similar for all six species. Therefore, several metabolites may be produced at much higher levels if the growth media would have been specifically optimized for a specific organism or metabolite production process. Depending on the carbon source, ethanol as well as acetic and lactic acid is produced by all tested bacteria and yeasts. In *T. reesei *only very few metabolites were produced above the indicated threshold, whereas *A. niger *is producing a wide variety of organic acids

**Table 4:** Extracellular metabolites formed in concentrations in excess of 0.1 g/l fermentation broth found in controlled fermentations of test organisms

Production host/carbon source	Alcohols and lactones	Organic acids	Amino acids/intermediates
*E. coli*/glucose	ethanol	**acetic**, **formic**, lactic, orotic, succinic, pyruvic	Glutamic, α-ketoglutaric acid, 2,3-dihydroxy-3-methylbutanoic acid

*E. coli*/xylose	ethanol	**acetic**, **formic**, lactic, propionic, succinic, pyruvic, orotic	2, 3-dihydroxy-3-methylbutanoic acid

*E. coli*/glycerol	ethanol	**acetic**, **formic**, propionic, succinic	glutamic, putrescine, 2, 3-dihydroxy-3-methylbutanoic acid

*C. glutamicum*/glucose	ethanol	**lactic**, succinic, acetic	glutamic

*C. glutamicum*/glycerol	ethanol	acetic, formic	-

*S. cerevisiae*/glucose	**ethanol**	**acetic**, **lactic**, malic, pyruvic, citric	-

*S. cerevisiae*/glycerol	**ethanol**	acetic, propionic, isobutyric, lactic, isovaleric	-

*P. stipitis*/glucose	ethanol	**pyruvic**, malic, acetic	-

*P. stipitis*/xylose	**ethanol**	citric, acetic, lactic	-

*P. stipitis*/glycerol	ethanol	lactic, citric, acetic	-

*T. reesei/*glucose	mannitol	malic	-

*T. reesei/*xylose	-	-	-

*A. niger/*glucose	**gluconic acid lactone**, ethanol, erythritol, glycerol	**gluconic, oxalic**, citric, malic, lactic, succinic, acetic, propionic	-

*A. niger/*xylose	arabitol, glycerol, mannitol	**malic, citric**, oxalic, propionic, succinic, acetic, pyruvic	-

*A. niger/*glycerol	-	**oxalic, propionic, succinic, malic**, isocitric, pyruvic citric	-

Products produced in excess of 1 g/l fermentation broth are in bold print. The products are listed in order of abundance.

### Fermentation performance on real life feedstocks

Hydrolysates of different biomass feedstocks as well as glycerol from biodiesel production were evaluated as fermentation feedstocks in shake-flask fermentation experiments. After hydrolysis, all lignocellulosic feedstocks were diluted to have similar glucose concentrations. Concentrations of xylose, arabinose, furfural, HMF and acetate are shown in Table 5. Biomass concentrations (g/l) were determined at the end of the cultures. In addition, glucose consumption was determined. For comparison, the latter parameter was expressed as the time point at which 50% of the initial glucose was consumed (Table 6).

**Table 5:** Concentrations (g/l) of several components present in final fermentation media generated from feedstock hydrolysates.

Feedstock (hydrolysis method)	xylose	arabinose	furfural	HMF	acetic acid
Sugar cane bagasse (EH)	8.3	0.6	0	0	2.1

Sugar cane bagasse (AH)	6.2	0.8	0.41	0.07	2.4

Wheat straw (EH)	6.6	1.1	0	0	0.9

Wheat straw (AH)	5.7	1.0	0.27	0.06	1.3

Corn stover (AH)	5.7	1.7	0.51	0.1	2.3

Willow wood (AH)	5.4	0.50	0.50	0.14	2.2

In all cases the hydrolysates were diluted to attain a glucose concentration of 15 g/l. AH = acid hydrolysis, EH = enzymatic hydrolysis.

**Table 6:** Growth on and utilization of real life feedstocks.

Feedstock (hydrolysis method)	*E. coli*	*C. glutamicum*	*S. cerevisiae*	*P. stipitis*	*T. reesei*	*A. niger*
	Biomass concentration (g/l) at the end of fermentation					

Sugar cane bagasse (EH)	7.4	12.4	1.2	10.5	3.8	4.9

Sugar cane bagasse (AH)	1.6	3.1	3.8	4.2	7.9	5.5

Wheat straw (EH)	1.1	2.5	3.7	7.3	4.6	6.8

Wheat straw (AH)	6.5	4.6	4.2	6.3	7.6	5.4

Corn stover (AH)	2.1	4.1	3.7	5.2	6.3	5.4

Willow wood (AH)	0	0	3.1	6.6	6.5	5.8

Glycerol from biodiesel	0	0	0.3	3.5	6.7	5.8

	Time (h) after which > 50% of the initial glucose/glycerol was consumed					

Sugar cane bagasse (EH)	20	20	15	20	120	100

Sugar cane bagasse (AH)	30	30	15	30	120	90

Wheat straw (EH)	20	20	15	20	120	90

Wheat straw (AH)	20	30	15	20	90	70

Corn stover (AH)	15	30	15	30	120	90

Willow wood (AH)	-	-	15	30	120	90

Glycerol from biodiesel	-	-	-	> 50*	120	120

AH = acid hydrolysis, EH = enzymatic hydrolysis.

*cultivation was terminated after 50 hours.

These results show that fungi can grow on all tested lignocellulosic feedstock hydrolysates. Bacteria did not grow on willow wood hydrolysate and glycerol from biodiesel. The latter feedstock was also not utilized by *S. cerevisiae *and poorly by *P. stipitis*. Remarkably, *T. reesei *grew very well on crude but not on pure glycerol (see Table 1). Maybe this is due to impurities in the crude glycerol that enable spore germination, which is a prerequisite for growth. Moreover, the high sodium chloride concentrations in the crude glycerol could have inhibited growth of *E. coli*, *C. glutamicum *and *S. cerevisiae*. In those cases where the strains did grow on crude feedstocks, they have similar glucose and glycerol consumption properties as found on pure substrates. Biomass concentrations for organisms that are known to co-utilize C5 sugars were higher on the crude feedstocks (see Table 6), most probably due to the consumption of the xylose and/or arabinose present in the medium (Table 5).

## Discussion

Although the necessity in the use of cheap feedstock for the use of bioprocesses for commodity products such as biofuels has long been well appreciated [18,19], remarkably little research was carried out to evaluate the suitability of industrial production host strains to utilize these substrates. In particular no direct comparison of different hosts has been conducted. In this study, this lack of knowledge was addressed by comparing the performance of six commonly industrially used production organisms. To allow a relevant comparison, a unified synthetic minimal medium was designed suitable for all species. However, it must be noted, that the yeasts *S. cerevisiae *and *P. stipitis *still did not grow on this medium without the supplementation of a vitamin solution and *C. glutamicum *did not grow without the supplementation of biotin (Methods). In this respect, both filamentous fungi and *E. coli *are less demanding which gives them an advantage in industrial fermentations, where expensive vitamin solutions are unlikely to be added. Moreover, growth conditions such as dissolved oxygen, pH and temperature were also kept constant in all controlled fermentations, which might have had an effect on some observations that were made.

The goal of this study was to compare the performance of six microorganisms by submitting them to growth conditions that they encounter in a second-generation production process, i.e. mixture of sugars, inhibitors, extreme pH etc. as well as comparing their performance on real-life feedstocks. Data generated from several small-scale and controlled fermentations were used to rank the microorganisms by relative performance. In order to establish an unbiased basis for the selection of microbial production hosts, we have scored the various criteria evaluated in this study. Scores were categorized from 1 (weakest performance) to 4 (best performance). On the basis of a lower weighting of the parameter, in some cases scores were categorized from 1 (weakest performance) to 2 (best performance). Zero (0) scores were given if the test organism did not grow under the scored condition. The accumulated results are shown in Table 7. As it was not possible to accurately determine the growth rate in crude feedstock cultures, in Table 7 only the related carbon source uptake rate for both pure and crude carbon sources was scored. *A. niger *and *P. stipitis *scored highest among the test organisms. It should, however be noted that in this study wild-type host strains were used. Obviously for each specific species, industrial host strains with improved characteristics could be developed or already available. No clear trend could be observed whether the hydrolysis method has an influence on the biomass concentration. The concentrated acid hydrolysis method in which the use of hydrolytic enzymes is completely avoided [11] seems suitable for producing fermentable hydrolysates for most of the tested cases. Although the enzymatic hydrolysis method produced less inhibitors furfural and HMF (Table 6), these inhibitors may still have an impact on final biomass concentration.

**Table 7:** Benchmarking of properties relevant for selection of microbial production hosts

Scoring	Performance parameter	*S. cerevisiae*	*P. stipitis*	*A. niger*	*T. reesei*	*E. coli*	*C. glutamicum*
0, 1	Vitamin requirement	0	0	1	1	1	0
0, 1, 2	Growth on xylose	0	2	2	2	2	0
0, 1, 2	Growth on glucose	2	2	2	2	2	1
0, 1, 2	Growth on arabinose	0	0	2	0	2	0
0, 1, 2	Growth on galactose	1	2	0	0	2	1
0, 1, 2	Growth on mannose	2	2	2	2	2	1
0, 1, 2	Growth on glycerol	1	2	2	0	2	1
**TOTAL medium versatility**		**6**	**10**	**11**	**7**	**13**	**4**

C-source uptake rate							
0, 1,2,3,4 *	Glucose	4	2	1	1	4	3
	Xylose	0	4	1	1	4	0
	Glycerol	1	2	1	0	3	3

Biomass yield							
0, 1,2,3,4 **	Glucose	1	1	3	2	2	3
	Xylose	0	1	3	2	2	0
	Glycerol	4	1	2	0	3	1

**TOTAL fermentation parameters**		**10**	**11**	**11**	**6**	**18**	**10**

0,1,2	pH <4	1	1	2	2	0	0
0,1,2,3,4	acetic acid resistance	2	2	4	4	2	3
0,1,2,3,4	HMF resistance	2	3	4	4	3	3
0,1,2,3,4	furfural resistance	2	2	2	2	2	1
0,1,2,3,4	NaCl resistance	1	2	4	4	2	2
0,1,2	MgSO_4 _resistance	2	2	2	2	1	2
	
**TOTAL inhibitors**		**10**	**12**	**18**	**18**	**10**	**11**

C-source utilization							
0, 1,2 ***	Sugar cane bagasse (EH)	2	2	1	1	2	2
	Sugar cane bagasse (AH)	2	2	1	1	2	2
	Wheat straw (EH)	2	2	1	1	2	2
	Wheat straw (AH)	2	2	1	1	2	2
	Corn stover (AH)	2	2	1	1	2	2
	Willow wood (AH)	2	2	1	1	0	0
	Glycerol from Biodiesel	0	2	1	1	0	0

Biomass concentration							
0, 1,2,3,4 ****	Sugar cane bagasse (EH)	1	4	2	2	3	4
	Sugar cane bagasse (AH)	2	2	3	3	1	2
	Wheat straw (EH)	2	3	3	2	1	2
	Wheat straw (AH)	2	3	3	3	3	2
	Corn stover (AH)	2	3	3	3	2	2
	Willow wood (AH)	2	3	3	3	0	0
	Glycerol from Biodiesel	0	2	3	3	0	0

**TOTAL feedstock versatility**		**23**	**34**	**27**	**26**	**20**	**22**

**TOTAL score**		**49**	**67**	**67**	**57**	**61**	**47**

0 = no growth, 1-4 or 1-2 = low to high score on performance parameters indicated.

* C-source uptake rate [g/g/h]: no growth = 0, <0.2 = 1, <0.6 = 2, <1.5 = 3, >1.5 = 4

** Biomass yield [g/g]: no growth = 0, <0.15 = 1, <0.3 = 2, <0.45 = 3; >4.5 = 4

*** C-source utilization, time [h] after which > 50% of the initial glucose/glycerol was consumed: no growth = 0, >70 = 1, >30 = 2

**** Biomass concentration [g/l]: <0.5 = 0, <2.0 = 1, <5.0 = 2, <10.0 = 3, 10.0 = 4

## Conclusion

Until now, attempts to improve host organisms are always aimed to improve one property at a time, such as increasing the product yield or making the strain more resistant against fermentation stress. The present benchmarking approach evaluated the basic performance of a production organism in controlled fermentations, use of carbon source, and resistance to inhibitors. There were clear differences in carbon source utilization by the test organisms. Of the most important monosaccharides in lignocellulosic feedstocks, xylose is not readily fermented by *S. cerevisiae *and *C. glutamicum*, whereas arabinose is not utilized by *P. stipitis*. In general, filamentous fungi are less susceptible to inhibitors from lignocellulosic hydrolysates than the other test organisms making them more robust for crude feedstock hydrolysate utilization.

## Methods

### Strains and culture conditions

In this study *Saccharomyces cerevisiae *S288C (ATCC 26108), *Pichia stipitis *CBS 6054 (ATCC 58785), *Aspergillus niger *ATCC 1015, *Trichoderma reesei *QM9414 (ATCC 26921), *Corynebacterium glutamicum *ATCC 13032 and *Escherichia coli *MG1655 (ATCC 47076) were used. All growth experiments were performed in a synthetic minimal medium containing (per litre of demineralised water) 8 g NH_4_Cl, 0.5 g (NH_4_)_2_SO_4_, 0.3 g MgCl_2_.6H_2_O, 40 mg EDTA, 2 mg ZnSO_4_.7H_2_O, 1 mg CaCl_2_.2H_2_O, 15 mg FeSO_4_.7H_2_O, 0.2 mg Na_2_MoO_4_.2H_2_O, 2 mg CuSO_4_.5H_2_O, 0.4 mg CoCl_2_.6H_2_O, and 1 mg MnCl_2_.4H_2_O. The initial pH was adjusted with 0.75 M K_2_HPO_4 _and 0.75 M NaH_2_PO_4 _to 4.5 for *A. niger *and *T. reesei*, to 5.0 for *S. cerevisiae *and *P. stipitis *and to 7.0 for *E. coli *and *C. glutamicum*. *S. cerevisiae *and *P. stipitis *cultures were supplemented with 0.05 mg/l biotin, 1.0 mg/l calcium pantothenate, 1.0 mg/l nicotinic acid, 25 mg/l myo-inositol, 1.0 mg/l thiamine-HCl, 1.0 mg/l pyridoxol-HCl and 0.2 mg/l *para*-aminobenzoic acid. The *C. glutamicum *cultures were supplemented with 0.05 mg/l biotin.

### Carbon source versatility

To analyse carbon source versatility all strains were cultivated in 15 ml test tubes containing 3 ml synthetic minimal medium supplemented with glucose, xylose, arabinose, mannose, galactose, or glycerol at a concentration of 40 g/l. Filamentous fungal cultures (inoculated from spores) were incubated at 25°C, yeasts and *C. glutamicum *at 30°C and *E. coli *at 37°C. Growth was monitored by visual inspection after 96 h of incubation and compared to a cultivation in the absence of carbon source.

### Controlled fermentations

For host comparison under controlled fermentation conditions all strains were grown in batch fermentations in a Bioflo 3000 (New Brunswick Scientific, Edison, New Jersey) bioreactor with 2 litres of synthetic minimal medium containing 40 g/l glucose, xylose, or glycerol as carbon source. The cultures were inoculated with 5% (v/v) of precultures grown overnight on the same medium. The dissolved oxygen concentration was maintained at 20% saturation by automatic adjustment of the stirrer speed. A constant pH (pH = 4.5 for *A. niger *and *T. reesei*; pH = 5.0 for *S. cerevisiae *and *P. stipitis*; pH = 7.0 for *E. coli *and *C. glutamicum*) was maintained by automatic titration with 2 M KOH and 1 M H_2_SO_4_. Samples were taken from the bioreactors during the course of the fermentation. The biomass concentration (cell dry weight) was determined according to [20] and the glucose, xylose and glycerol concentrations were determined by HPLC [21,22]. Based on the data obtained, for each culture growth rate, biomass yield and carbon source uptake rate were calculated. The fermentations were stopped after carbon source depletion.

### Inhibitor screening

The resistance against inhibitors was monitored by cultivating the six microorganisms in the synthetic minimal medium containing 40 g/l glucose as major carbon source and different concentrations of inhibitor (furfural and HMF at 0.5 to 5 g/l, acetate at 1 to 20 g/l, sodium chloride at 5 to 100 g/l and magnesium sulphate at 20 to 80 g/l). Growth inhibition was followed by measuring the OD_600 _in the case of *E. coli*, *C. glutamicum*, *S. cerevisiae *and *P. stipitis *and by following CO_2 _levels in the exhaust gas phase in for the filamentous fungi *A. niger *and *T. reesei*. To correlate the two different measurement methods, CO_2 _levels were also measured for *S. cerevisiae*. The OD_600 _measurements were performed using a Bioscreen C MBR (Thermo Electron, Hanau, Germany) according to the manufacturer's instructions. The growth rate was calculated using EZExperiment, the software accompanying the Bioscreen C MBR. CO_2 _levels were measured during the cultivation using the Micro-Oxymax™(Columbus Instruments, Columbus, Ohio).

### Exometabolome analysis

From all controlled fermentations, a sample was taken at the late log-phase for exometabolome analysis. To these samples phenylalanine-d5 (Cambridge Isotope Laboratories, Andover, MA) was added as an internal standard. This sample was filtered through a 0.2 μm membrane filter (Sartorius, Göttingen, Germany) and divided in two aliquots, one for GC- and one for LC-MS analysis. The LC-MS sample was deproteinized by filtration using a Satorius Centrisart I 10 kDa filter centrifuged at 2000 g and -4°C for 16 hours. Subsequently, both the GC- and LC-MS samples were lyophilized.

The exometabolomes of these samples were analyzed by applying oximation silylation (OS)-GC-MS and ion pair (IP)-LC-MS Both methods together give a coverage of > 90% for microbial metabolomes [16]. For OS-GC-MS analysis, lyophilized samples were derivatized by oximation using ethoxyamine hydrochloride followed by silylation with N-trimethyl-N-trimethylsilylacetamide [15]. Subsequently, the derivatized samples were analyzed by electron impact GC-MS-analysis [16]. For IP-LC-MS analysis, lyophilized samples were dissolved in methanol/water (1:3 v/v) and separated on a reversed-phase column using an eluent containing hexylamine as the ion-pairing agent [14]. Compounds were detected by electrospray ionization using a linear ion trap mass spectrometer [15]. The concentration of the compounds was estimated on the basis of the response factor of the mass detector [16].

In addition, concentrations of ethanol [21] and acetate [22] were determined in the freshly harvested samples by HPLC.

### Fermentation of hydrolysates

Sugar cane bagasse, wheat straw, corn stover and willow wood were a kind gift from Zilor (Brazil), Oostwaardhoeve (the Netherlands), University of Cape Town (South Africa) and Oostwaardhoeve, respectively. Crude glycerol containing 10% sodium chloride, a co-product from biodiesel production, was purchased from Cremer Oleo (Hamburg, Germany). The lignocellulosic feedstocks were hydrolyzed using both mild acid thermal pre-treatment followed by enzymatic hydrolysis (thermozyme) [9] and concentrated acid hydrolysis (Biosulfurol) [10,11] methods. Thermal mild acid pretreatment was carried out as follows: 2.5 kg dried biomass was mixed with 15 kg water and 0.003 kg sulphuric acid and heated 60 minutes in a 25 l autoclave (manufactured by Andreas Hofer, Germany, maximum temperature 350°C ) at 160°C. After cooling down the pH was set at 5 using a solution of 1 M KOH. 300 ml cellulase/cellobiase (GC220 from Genencor, The Netherlands) and 40 ml Novozyme 188 (Novozyme, The Netherlands) was added to 15 litre slurry of pre-treated biomass and incubated 24 hours at 50°C in a stirred 20 litre reactor at pH 5. For further use the remaining solids were removed by centrifugation at 8500 rpm (Sorvall Evolution with SLC-6000 rotor). The supernatant was filter-sterilized (0.2 μm cellulose membrane) and stored at -20°C.

Pre-treatment of biomass using concentrated acid was carried out as follows: 200 g of dried biomass was transferred to a 2 l Schott bottle, 336 ml of 72% sulphuric acid (sulphuric acid: pulp = 2:1 (w/w)) was added and the solution was mixed on a roller bank for 24 hours at 30°C. The pre-treated biomass was subsequently poured in a 5 l Schott bottle, the 2 l Schott bottle was washed with a total of 2400 ml water and the resulting liquid transferred to the 5 l bottle. The 5 l bottle was covered with aluminium foil and incubated at 95°C for 3 hours. The bottle was not fully closed to allow the formed gases to escape. Neutralization and sulphate removal was carried out as follows: 300 g/l CaOH_2 _was added to the suspension in the 5 l Schott bottle until a pH of 5.5-6.5 was reached, at which point the fluid thickened to a semi-solid slurry. This slurry was then centrifuged for 20 min. at 6000 rpm using a Sorvall Evolution RC Superspeed centrifuge (Thermo Fisher Scientific Inc., Breda, The Netherlands) to separate the CaSO_4 _and other solids (lignin) from the liquid containing the sugars released from the biomass. The supernatant containing the sugars was removed and stored at -20°C until it was used for fermentation.

The glucose concentration of all hydrolysates was analyzed enzymatically (Horiba ABX, Montpellier, France) using a COBAS MIRA Plus autoanalyzer (Roche Diagnostic Systems, Basel, Switzerland). The hydrolysates were subsequently diluted to 15 g/l glucose while adding the same amounts of minerals as used in the synthetic minimal medium described above. Xylose, arabinose, furfural, HMF and acetate concentrations were determined by HPLC [21,22]. The concentration of crude glycerol in minimal medium was 40 g/l. 50 ml cultures in 250 ml baffled shake flasks were inoculated with 5% (v/v) of precultures of all strains grown overnight in the glucose based minimal medium and incubated for 168 h in a orbital shaker (150 rpm) under conditions used above for each of the strains. Samples were taken during the course of the cultivations and analyzed for residual glucose, xylose, and glycerol and biomass [21,22].

## Competing interests

The authors declare that they have no competing interests.

## Authors' contributions

KR participated in the design of experiments, coordinated the study and drafted the manuscript. HJJVB carried out the hydrolysis, fermentation and analysis experiments. KMO participated in the design of the experiments and carried out fermentation experiments. JWVG T participated in the design of experiments and helped to draft the manuscript. PP participated in the design of experiments and helped to draft the manuscript. MJVDW conceived the study, and participated in its design and coordination and helped to draft the manuscript. All authors read and approved the final manuscript.
